# ONE-TWO PUNCH OF HIGH WAGES AND EMPOWERING PRACTICES FOR MAINTAINING CARE NETWORKS IN NURSING HOMES

**DOI:** 10.1093/geroni/igz038.2068

**Published:** 2019-11-08

**Authors:** Katherine Kennedy, John R Bowblis, Katherine M Abbott

**Affiliations:** 1 Miami University, Department of Sociology and Gerontology, Oxford, Ohio, United States; 2 Miami University, Department of Economics, Farmer School of Business, Oxford, Ohio, United States; 3 Department of Sociology and Gerontology, Scripps Research Fellow, Miami University, 45056, Ohio, United States

## Abstract

Stabilizing certified nursing assistant (CNA) employment is necessary for maintaining care networks and providing high quality of care for nursing home (NH) residents. This study’s objective was to examine the relationship of high wages and empowerment practices on CNA retention. We used the 2015 Ohio Biennial Survey to construct a facility-level dataset of 547 NHs and estimated multivariable linear regressions. NHs that provided both high wages and high empowerment were associated with a 12.95 percentage-point improvement in the CNA retention rate (SE = 4.53, t-value = 2.86, p = 0.0045). High wages and a high empowerment score did not have significant effects individually (p > .05). Retention rates were similar between NHs that lacked high wages and scored low on the empowerment scale, and NHs that provided one at a high level but not the other. Implications for better retaining CNAs require multiple empowerment practices combined with high hourly wages.

